# Non-Culture Diagnostics for Invasive Candidiasis: Promise and Unintended Consequences

**DOI:** 10.3390/jof4010027

**Published:** 2018-02-19

**Authors:** Cornelius J. Clancy, M. Hong Nguyen

**Affiliations:** 1 Division of Infectious Diseases, University of Pittsburgh, Scaife Hall 867, 3550 Terrace St., Pittsburgh, PA 15261, USA; 2 Department of Medicine, University of Pittsburgh, Pittsburgh, PA 15261, USA; mhn5@pitt.edu

**Keywords:** *Candida*, candidiasis, candidemia, diagnostic, T2Candida, polymerase chain reaction, 1,3-β-d-glucan, Bayesian

## Abstract

Blood cultures are positive for *Candida* species in < 50% and < 20% of hematogenously disseminated and intra-abdominal candidiasis, respectively. Non-culture tests such as mannan, anti-mannan antibody, *Candida albicans* germ tube antibody (CAGTA), 1,3-β-d-glucan (BDG), the T2Candida nanodiagnostic panel, and polymerase chain reaction (PCR) are available for clinical use, but their roles in patient care are uncertain. Sensitivity/specificity of combined mannan/anti-mannan, BDG, T2Candida and PCR for candidemia are ~80%/80%, ~80%/80%, ~90%/98%, and ~90%/90%, respectively. Limited data for intra-abdominal candidiasis suggest CAGTA, BDG sensitivity/specificity of ~65%/75% and PCR sensitivity of ~85–90%. PCR specificity has varied widely for intra-abdominal candidiasis (33–97%), and T2Candida data are lacking. Tests will be useful if restricted to cases in which positive and negative predictive values (PPVs, NPVs) differ in a clinically meaningful way from the pre-test likelihood of invasive candidiasis. In some patients, PPVs are sufficient to justify antifungal treatment, even if blood cultures are negative. In most patients, NPVs of each test are excellent, which may support decisions to withhold antifungal therapy. If test results are not interpreted judiciously, non-culture diagnostics may have unintended consequences for stewardship and infection prevention programs. In particular, discrepant non-culture test-positive/culture-negative results may promote inappropriate antifungal treatment of patients who are unlikely to have candidiasis, and lead to spurious reporting of hospital-acquired infections. In conclusion, non-culture *Candida* diagnostics have potential to advance patient care, but this promise will be realized only if users understand tests’ strengths and limitations, and plan proactively for how best to employ them at their hospitals.

## 1. Introduction

*Candida* species are among the most common causes of nosocomial bloodstream infections, and of invasive infections in intensive care units (ICUs). Approximately 50% of primary candidemia results in deep-seated infections due to hematogenous seeding. The most common manifestations of deep-seated candidiasis are intra-abdominal infections such as peritonitis or abscesses. Intra-abdominal candidiasis typically stems from gastrointestinal tract disruption or an infected peritoneal catheter. Therefore, invasive candidiasis comprises three disease entities: (1) Candidemia in the absence of deep-seated candidiasis; (2) candidemia associated with deep-seated candidiasis; and (3) deep-seated candidiasis in the absence of candidemia [1].

Mortality rates among patients with candidemia range from ~20–40% [2,3]. At best, outcomes of candidemia have improved marginally over the past 25 years, despite advances in ICU practice and the introduction of new azole and echinocandin antifungals [4]. Intra-abdominal candidiasis manifests most commonly as abscesses and/or peritonitis; mortality ranges from 20–80%, depending on the disease manifestations [3]. For both candidemia and intra-abdominal candidiasis, the institution of timely antifungal therapy and source control are crucial determinants of good outcomes [2,3]. However, definitive treatment often is delayed due to the relative insensitivity of microbiologic cultures, the gold standard diagnostic [1]. Data from autopsy studies suggest that the sensitivity of blood cultures in cases of hematogenously disseminated candidiasis is < 50%. Blood cultures are positive for *Candida* in only ~5–20% of patients with intra-abdominal candidiasis. The sensitivity of deep tissue cultures for invasive candidiasis is also ~50%, and collection of these samples is often dangerous or contra-indicated in hospitalized patients. Cultures are further limited by slow turn-around times (typically requiring 2–3 days for growth to be evident), and the fact that they often turn positive late in the course of infection. For these reasons, the development and validation of non-culture diagnostic tests for candidemia, intra-abdominal candidiasis, and other types of invasive candidiasis is a top medical priority [1,4].

Several non-culture diagnostics for invasive candidiasis are now available for use as adjuncts to cultures. Mannan and anti-mannan IgG tests (Platelia *Candida* Ag-Plus and Ab-Plus, Bio-Rad, Marnes-la-Coquette, France; Serion Mannan Kit, Serio GmbH, Wurzburg, Germany), and *C. albicans* germ tube antibody assays (CAGTA; Vircell Kit and VirClia IgG Monotest, Grenada, Spain) are employed at many European centers. The tests are not widely used in North America, nor are they cleared by the United States Food and Drug Administration (FDA). The FDA has cleared a 1,3-β-d-glucan (BDG) assay (Fungitell, Associates of Cape Cod, East Falmouth, MA, USA) and the T2Candida nanodiagnostic panel (T2 Biosystems, Lexington, MA, USA) for the diagnosis of invasive fungal infections and candidemia, respectively. There are no FDA-cleared polymerase chain reaction (PCR) assays for *Candida*, but commercial and in-house tests are widely available. The objectives of this paper are to provide updates on new data from studies of non-culture tests for candidemia and intra-abdominal candidiasis, discuss how these tests might improve patient care, and consider unintended consequences of testing for stewardship and infection prevention programs. We will focus on testing of whole blood or blood fractions, since data for other types of samples are scant or absent.

## 2. Non-Culture Tests for Invasive Candidiasis

### 2.1. Mannan, Anti-Mannan Antibody, and C. albicans Germ Tube Antibody

In a meta-analysis of 14 studies, the sensitivity and specificity of mannan and anti-mannan IgG antibody for invasive candidiasis were 58% and 93%, and 59% and 86%, respectively [5]. Sensitivity and specificity for a combined mannan/anti-mannan assay were 83% and 86%, respectively, with best performance in patients with *C. albicans*, *C. glabrata* or *C. tropicalis* infections. Most data are for the diagnosis of candidemia. In a multi-center study of intra-abdominal and other deep-seated candidiasis, mannan and anti-mannan antibody demonstrated poor sensitivity (40% and 25%, respectively). There is less experience with CAGTA, which detects responses against a hyphal protein (Hwp1) expressed during tissue invasion and biofilm formation [6]. The sensitivity and specificity of CAGTA for invasive candidiasis have ranged from 42–96% and 54–100%, respectively, in different reports [6,7,8,9]. In one study, CAGTA sensitivity was 69% for candidemia complicated by deep-seated candidiasis, compared to only 5% for candidemia in the absence of deep-seated candidiasis [6]. Across several studies, sensitivity and specificity of CATGA for deep-seated candidiasis ranged from 53–73% and 54–80%, respectively [7,8,9]. Sensitivity of CATGA may be lower for infections caused by *C. tropicalis* than other *Candida* species.

### 2.2. BDG

1,3-β-d-glucan is a cell wall constituent of *Candida* and most other pathogenic fungi, excluding *Cryptococcus* and *Mucorales*. As such, BDG assays do not identify *Candida* species, or distinguish between *Candida* and other fungi. Commercial tests, including Fungitell, are indirect colorimetric or turbidmetric assays of serum that quantify BDG-mediated activation of a horseshoe crab coagulation cascade. The pooled sensitivity and specificity of BDG for invasive candidiasis were ~75–80% and ~80%, respectively, in meta-analyses [10,11,12]. This performance is based overwhelmingly upon studies of candidemia. More recently, investigators have explored Fungitell for the diagnosis of deep-seated infections, in particular intra-abdominal candidiasis [7,8,13,14]. Fungitell sensitivity and specificity ranged from 56–77% and 57–83%, respectively (median: ~65% and ~75%).

BDG performance is better if positivity is defined by two consecutive results, rather than a single result [15]. Sensitivity may be reduced for *C. parapsilosis* infections [16]. Factors associated with false-positive BDG results are common among hospitalized patients, including *Candida* or mold colonization, receipt of human blood products or certain β-lactam antibiotics, hemodialysis or hemofiltration, presence of some Gram-positive bacteria, cellulose dressings, enteral nutrition, mucositis, and disruptions of GI tract integrity [1]. Since culture is a suboptimal gold standard, specificity measurements are a major uncertainty in any study of *Candida* diagnostics, especially if controls are at-risk for invasive candidiasis. Another limitation of Fungitell is that kits consist of one-time use, 96-well trays, which means that hospitals will generally perform batch testing no more frequently than once or twice a week. This delay limits the utility of the test for rapid initiation of antifungal agents. Negative tests, however, may still be useful in decisions to discontinue antifungal therapy [17,18].

### 2.3. T2Candida Panel

T2Candida uses an automated instrument platform (T2Dx) to detect *Candida* directly within whole blood in K_2_ EDTA vacutainer collection tubes. T2Dx lyses red blood cells, concentrates *Candida* cells and cellular debris, lyses cells by mechanical bead-beating, and amplifies DNA using a thermostable polymerase and primers for ribosomal DNA intervening transcribed spacer region 2 [19,20]. Amplified product is detected by amplicon-induced agglomeration of supermagnetic particles and T2 magnetic resonance. Results are reported as positive or negative for *C. albicans/C. tropicalis*, *C. glabrata/C. krusei,* and *C. parapsilosis*, groupings that are based on typical antifungal susceptibility patterns. These species account for >95% of invasive candidiasis at most hospitals [21], but microbiology can differ by center and clinicians must be aware of local data [22]. The limit of detection depending on species is 1–3 CFU/mL, which is superior to that generally reported for PCR assays [1,20].

FDA clearance of T2Candida was based on data from the multi-center DIRECT trial, which included >1500 control patients with *Candida*-negative blood cultures, 6 patients with *Candida*-positive blood cultures, and 250 contrived blood specimens spiked with *C. albicans*, *C. glabrata*, *C. parapsilosis*, *C. tropicalis* or *C. krusei* at concentrations ranging from 1–100 CFU/mL [20]. Per-patient sensitivity and specificity were 91% and 98%, respectively. The mean time to *Candida* detection and species identification was 4.4 ± 1.0 h, which suggests that results will be available to clinicians to impact decisions on both treatment initiation and discontinuation. In the follow-up, multi-center DIRECT2 trial, T2Candida sensitivity was 89% in 36 patients at the time of positive blood cultures for *Candida* [19]. Among 152 patients with prior candidemia (i.e., within 1–6 days), T2Candida was significantly more likely to be positive than concurrently collected blood cultures (45% vs. 24%). The higher positivity for T2Candida compared to blood cultures was driven by performance among patients receiving antifungal therapy.

At present, there are no data on T2Candida performance for types of invasive candidiasis other than candidemia. Invalid T2Candida results were obtained for 7–9% of thawed whole blood samples in DIRECT and DIRECT2; rates using fresh blood samples in routine clinical practice are undefined. Other uncertainties for T2Candida that are shared with BDG and PCR assays include the clinical significance of discrepant T2Candida-positive/culture-negative results, the precise effects of antifungal treatment on assay performance, the kinetics and prognostic value of serial test results, and the test’s role in guiding patient care.

### 2.4. PCR Assays

Even more so than for other non-culture diagnostics, interpretation of PCR data is complicated by heterogeneity of assays and study design. Multiple commercial and in-house tests, including multiplex formats capable of detecting other fungi and/or bacteria, have been investigated. In a meta-analysis of 54 studies that included almost 5000 patients tested by blood-based PCR, pooled sensitivity and specificity for proven or probable invasive candidiasis (candidemia predominantly) vs. at-risk controls were 95% and 92%, respectively [23]. Pooled sensitivity and specificity for proven, probable or possible invasive candidiasis vs. at-risk controls were 73% and 95%, respectively. Data for infections other than candidemia are limited. In several recent studies, the sensitivity of PCR assays for intra-abdominal candidiasis ranged from 86–91%, but specificity varied widely, from 33% to 70% to 97% [7,8,13]. In the PCR meta-analysis, higher sensitivity was observed with whole blood rather than serum, panfungal rRNA or P450 genes as targets, *Candida*- or fungal-specific assays rather than broader multiplex assays, and in vitro detection limits ≤10 CFU/mL. There was a trend toward lower specificity among controls who were colonized by *Candida*.

As for T2Candida, multiplex PCR tests generally target the five most common pathogenic *Candida* species. No PCR assay has been validated for diagnosing invasive candidiasis in multi-center studies, and there is no conclusive evidence that any commercial test is superior. Turn-around time for PCR will depend upon the type of assay being performed, and the work-flow and capacity in individual hospital labs. In general, PCR and T2Candida offer potential advantages over BDG in time to results, and by targeting *Candida* and providing species identification. BDG may offer advantages if other invasive fungal infections are also considerations.

## 3. Non-Culture Tests as Bayesian Biomarkers

Non-culture tests are not categorical diagnostics, but rather Bayesian biomarkers that assign a probability of infection [24,25]. Management decisions based on test results will be left to the best judgment of providers. The positive and negative predictive values (PPVs, NPVs) of a test are determined by sensitivity and specificity, and the patient’s pre-test likelihood of invasive candidiasis.

Pre-test likelihoods of candidemia and intra-abdominal candidiasis can be estimated in patients with signs of infection from data on disease prevalence in various clinical settings. Risk factors for candidemia are relatively common in hospitalized patients, including receipt of broad-spectrum antibiotics, intravenous access devices, total parenteral nutrition, mechanical ventilation, hemodialysis, diabetes mellitus, corticosteroids, neutropenia or neutrophil dysfunction, and *Candida* colonization. The prevalence of candidemia increases from <1% to ~10% as one moves from any patient in whom blood cultures are collected, to low-risk ICU patients, to more moderate-risk patients who are ICU residents for ≥ 4 days or who are in septic shock, to higher-risk ICU patients identified by clinical prediction scores (Table 1) [19]. Intra-abdominal candidiasis occurs in a subset of patients who, in addition to risk factors for candidemia, have predisposing GI tract or digestive system abnormalities. The prevalence of intra-abdominal candidiasis increases from ~5% to ~30% as one moves from low-to-moderate risk peritoneal dialysis patients with peritonitis, to high-risk patients with severe necrotizing pancreatitis or recurrent GI tract leaks (Table 2) [25,26,27,28,29].

In most patients in whom infection is suspected, the predominant type of invasive candidiasis should be apparent when a test is ordered, and anticipated PPVs and NPVs can be calculated (Table 1 and Table 2). Estimates of mannan/anti-mannan antibody, BDG and PCR PPVs and NPVs can be made more confidently than for candidemia than intra-abdominal candidiasis, since data are much more extensive. CAGTA antibody data specifically for candidemia are limited. T2Candida PPVs and NPVs can only be calculated for candidemia because sensitivity and specificity data are lacking for intra-abdominal candidiasis.

At low pre-test likelihoods of either candidemia or intra-abdominal candidiasis, PPVs and NPVs are extremely low and extremely high, respectively. As likelihoods increase, PPVs increase and NPVs decrease. For each type of patient at-risk for candidemia in Table 1, NPVs of mannan/anti-mannan antibody, BDG, T2Candida and PCR are exceptional (≥97%). Mannan/anti-mannan and BDG PPVs increases to ~30% for high-risk ICU patients who fulfill clinical prediction criteria for candidemia. PPVs for T2Candida and PCR are expected to be superior to BDG.

In patients at low risk for intra-abdominal candidiasis, the anticipated CAGTA and BDG NPV is strong (>98%). In higher-risk settings, however, values drop to ~80% (e.g., severe acute or necrotizing pancreatitis, high-risk GI surgery). CAGTA and BDG PPVs rise to ~50% among the highest-risk patients. Due to the highly disparate specificities reported thus far for PCR in diagnosing intra-abdominal candidiasis, it is not possible to estimate predictive values accurately. If specificity is only 33%, NPVs will be similar to those for BDG, but PPVs will not be significantly different from the pre-test likelihood. If specificity is 70%, NPVs should improve and PPVs should be comparable to those for BDG. If specificity is 97%, NPVs would be further improved; moreover, PPV would approach 50% in low-risk patients, and exceed 90% in highest-risk patients.

## 4. How Non-Culture Diagnostics Might Improve Patient Care

The threshold probability of invasive candidiasis that justifies antifungal treatment is not known. A number of studies in patients with hematologic malignancies, critical illnesses, and/or multiple risk factors for invasive fungal infections suggest that antifungal prophylaxis is beneficial if the baseline rate of disease is ≥ 15–30% [24,25]. Therefore, the target PPV and NPV for triggering empiric treatment of invasive candidiasis are likely to be in the 15–30% range and >85%, respectively. Based on these targets, non-culture tests are predicted to be most valuable in the clinical settings that appear within the boxes in Table 1 and Table 2. At some pre-test likelihood of candidemia, a given test is useful to a provider because a positive result increases the probability of disease above the 15–30% threshold, while a negative result virtually excludes the diagnosis. Given these considerations, it is readily apparent that none of the tests is likely to have value for diagnosing candidemia if ordered each time a blood culture is collected, since anticipated PPVs are ≤15% and NPVs are not significantly lower than the pre-test probability.

CAGTA and BDG are most likely to be useful for diagnosing intra-abdominal candidiasis within a window between lowest and highest risk groups. At the lowest pre-test likelihoods (e.g., < 5–10%), PPVs are probably insufficient to justify treatment, and negative results minimally reduce the probability of infection. In the highest risk patients, it is not clear that PPV of ~50% would have greater practical value for decision-making than knowing a pre-test likelihood of ~30%. At the same time, the anticipated NPV of ~80% means that clinicians must be willing to forego treatment despite a ~20% chance that disease is present. PCR would have no clinical utility if specificity for intra-abdominal candidiasis is only 33%. If specificity is 70%, PCR likely would be useful for more patients than CAGTA or BDG. If specificity is 97%, then the test may be useful in almost any patient at-risk for intra-abdominal candidiasis.

Table 1 and Table 2 provide a conceptual framework for interpreting non-culture test results. Of course, there are multiple other factors that providers must weigh as they use results to make treatment decisions for individual patients. Considerations such as number and types of risk factors for candidiasis, severity of illness, physical findings, imaging and lab data, and the possibility of alternative diagnoses may increase or decrease the pre-test likelihood of disease. Likewise, post-test probability may be influenced by the magnitude of results; two highly positive values are more compelling than a single borderline result. It is infeasible for clinicians to calculate precise running tallies of pre- and post-test likelihoods in each patient. Nevertheless, they can conceptualize probabilities qualitatively. Examples of qualitative evaluations that can guide decision-making are “my patient is reasonably likely to have invasive candidiasis, and a positive result significantly increases that possibility”, or “my patient has some risk factors for candidemia, but a negative result makes the disease extremely unlikely.”

A particularly challenging decision for clinicians is the NPV threshold at which they are comfortable withholding antifungal treatment in a given patient. Among patients at the highest risk for intra-abdominal candidiasis, for example, a negative result for an excellent PCR assay (85% sensitivity/97% specificity) would still leave a ~6% chance of infection. For an especially sick patient in whom an alternative diagnosis is not evident, treatment might be offered despite this low predictive value. In such a case, non-culture testing should not be performed, since results will not impact treatment decisions. Indeed, prior to ordering any diagnostic test, clinicians should pre-determine how results will be used in managing the patient.

In the end, the expectation is that non-culture diagnostics, employed rationally, will identify at least some patients with invasive candidiasis earlier than cultures, others with infections that are missed by cultures, and large numbers of patients in whom invasive candidiasis is extremely unlikely. Moving forward, studies are needed to establish that patient management and stewardship strategies exploiting these properties improve outcomes, reduce unnecessary antifungal usage, limit emergence of resistance, and are cost-effective. The roles of combination testing also merit careful investigation [8,17].

## 5. Unintended Consequences of Non-Culture Testing

While acknowledging the promise of non-culture diagnostics, it is important to anticipate unintended consequences of widespread testing. In particular, the use of non-culture tests has the potential to drive extra antifungal consumption, and lead to reporting of more hospital-acquired infections (HAIs). Discrepant results in which non-culture tests are positive and blood cultures are negative are likely to pose the greatest challenges.

### 5.1. Impact on Antifungal Consumption

Modeling studies of T2Candida have suggested that stewardship strategies exploiting strong NPVs can reduce antifungal usage and hospital costs, without compromising patient outcomes [45]. However, if discrepant non-culture positive/blood culture negative results are common at a given center, it is possible that more patients will be treated than at present, thereby offsetting potential reductions in antifungal consumption. Treating patients at a 15–30% PPV threshold may have benefit across a population, but it means that a large percentage of patients started on an antifungal agent will not have candidiasis. Extreme examples of how non-culture testing may fuel antifungal consumption are afforded by patients receiving mechanical circulatory support with left ventricular assist devices (LVAD). Patients with LVAD-associated candidemia are treated typically with a prolonged, primary course of an antifungal, followed by a suppressive regimen for the duration of time that the device remains in place. LVAD *Candida* infections often are associated with biofilms that may harbor latent yeast cells or lead to intermittent candidemia. Since non-culture tests are not necessarily dependent upon the presence of viable organisms, they are likely to be positive in at least some LVAD patients with negative blood cultures. Therefore, this population may significantly distort antifungal stewardship metrics at high-volume centers. Anticipated PPVs for LVAD patients with signs of infection are shown in Table 2. Even in best case scenarios with T2Candida, ~30% of positive results may not be associated with true candidemia.

### 5.2. Impact on Reporting of HAIs

The potential of non-culture test-positive/blood culture-negative results to increase the reporting of HAIs has profound implications for U.S. hospitals. To get payment from Medicare, hospitals are required to report data about certain HAIs to the Centers for Disease Control and Prevention’s National Healthcare Safety Network (NHSN), including central line-associated bloodstream infections (CLABSIs), catheter-associated urinary tract infections, surgical site infections, methicillin-resistant *Staphylococcus aureus* bacteremia, and *Clostridium difficile* infections [46]. In October 2014, the Centers for Medicare and Medicaid Services began reducing Medicare payments to hospitals that rank in the worst performing quartile for hospital-acquired conditions (HACs). For fiscal year 2018, the worst performing quartile is identified by calculating a total HAC score based on hospitals’ performance in six quality measures, five of which are rates of the NHSN HAIs listed above.

Among HAIs, non-culture *Candida* diagnostics are most relevant in identifying CLABSIs. NHSN definitions of CLABSI are built upon multiple criteria, including those for concepts that are relevant to all HAIs (e.g., “hospital-acquired”, “date of event”, “infection window period”, “present on admission”, etc.) and others that are more specific (e.g., laboratory confirmed bloodstream infection (LCBI), types of central lines, etc.). The starting point for identifying CLABSIs is identifying LCBIs. In defining LCBI, NHSN specifies the following: ”Patient of any age has a recognized pathogen, which is an organism not included on the NHSN common commensal list, identified from one or more blood specimens obtained by a culture or non-culture based microbiologic testing method (excluding organisms identified by testing on sera)” [47]. In this context, *Candida* species are not considered commensals. “A final laboratory report found in the medical record that identifies an organism is eligible for use in meeting the NHSN infection definition”, provided the test was “performed for purposes of clinical diagnosis and treatment” [48]. It does not matter if a “non-culture based microbiologic testing method” is FDA-cleared, not FDA-cleared or research-use only, or if results appear in the medical record with a research disclaimer (e.g., “This test is intended for investigational use only. The test results will not be used to diagnose your condition or to help your doctor determine how to treat your condition.”). The definition excludes testing that “was performed as part of active surveillance for carriage of organisms for the purposes of instituting or discontinuing isolation precautions.”

The criteria above do not treat non-culture diagnostics equally in identifying *Candida* CLABSIs. Infections identified by BDG are excluded because *Candida* are not detected specifically, and testing is performed on sera. Bloodstream infections detected by PCR and T2Candida using blood specimens fall within guidelines, but presumably those detected by serum PCR do not. Ambiguity in identifying *Candida* CLABSIs stems from the vague wording of criteria (e.g., a final laboratory report of a non-culture test is “eligible for use” in fulfilling a definition of CLABSI). Furthermore, assigning CLABSIs in individual patients is often complex, and dependent upon the clinical judgment of infection practitioners as they review laboratory results and other information in medical records. Given the probabilistic nature of non-culture diagnostics, how should discrepant results be interpreted in defining a *Candida* CLABSI? Assuming that other criteria for CLABSI are fulfilled in a given patient, infection prevention programs might pursue four basic strategies:

1. Report any case with a positive blood-based PCR or T2Candida result. This strategy certainly will overstate *Candida* CLABSI rates.

2. Use treatment decisions by clinicians in response to a positive blood-based PCR or T2Candida to identify CLABSI. This approach will accept provider bias.

3. Attempt to estimate PPV in each case. This strategy is likely beyond what is feasible for most infection prevention programs, and it will depend upon un-validated cut-off PPVs to define CLABSIs.

4. Do not report non-culture test positive/blood culture-negative cases, based on the concept that the biological and clinical significance of discrepant results is unknown at present. This strategy certainly will understate true CLABSI rates, but would not change the way most hospitals currently identify cases.

Given the stewardship and reimbursement implications of these issues, it is incumbent upon professional communities to engage with one another before non-culture testing is widespread to chart rational and reasonable paths forward.

## 6. Conclusions

Non-culture diagnostics for candidiasis have the potential to transform patient care, but they also present challenges to clinicians, laboratories and hospitals. The full value of these tests will be realized only if they are employed rationally, and targeted to particular patient populations and clinical questions. The best use of assays and interpretation of results at each center should be determined before testing is introduced. In the absence of such considerations, non-culture tests may be unhelpful or even deleterious.

## Figures and Tables

**Table 1:** Prevalence of candidemia in different populations, and anticipated PPVs and NPVs of non-culture tests.

Prevalence	Representative Patient (Reference)	^1^ BDG, Mannan/Anti-Mannan		^2^ T2Candida		^3^ PCR	
		PPV	NPV	PPV	NPV	PPV	NPV
**0.4%**	Any hospitalized patient in whom a blood culture is collected [20]	1%	99.9%	15%	>99.9%	3%	>99.9%
**1%**	Patient admitted to intensive care unit (ICU) [30,31]	4%	99.7%	31%	99.9%	8%	99.9%
**2%**	Patient with febrile neutropenia, baseline rate of candidemia prior to empiric antifungal treatment [32,33,34,35]	7%	99.5%	47%	99.8%		99.8%
**3%**	Patient with septic shock and > 3–7 days in ICU [30,36,37,38]	11%	99.2%	67%	99.7%	22%	99.7%
**5%**	Patient with left ventricular assist device and evidence of active infection [39,40]	17%	98.7%	70%	99.5%	32%	99.5%
**10%**	Patient fulfilling criteria of clinical prediction model for candidemia [41,42,43]	31%	97%	82%	99%	50%	99%

Sensitivity and specificity for candidemia for each test are estimated from studies cited in the text. ^1^ Sensitivity/specificity: 80%/80%; Data for CAGTA are more limited, but performance for the diagnosis of candidemia complicated by deep-seated candidiasis appears to be comparable to mannan/anti-mannan and BDG. ^2^ Sensitivity/specificity: 90%/98%; ^3^ Sensitivity/specificity: 90%/90%; PPV: Positive predictive value; NPV: Negative predictive value; PPVs and NPVs within the dark black lines signify patients in whom non-culture testing may have greatest clinical utility, assuming that antifungal treatment is justified at a threshold likelihood of invasive candidiasis of ≥~15–30%. For the patients indicated, a positive result is anticipated to move the likelihood of candidemia from below the threshold to above the threshold. At the same time, negative tests make candidemia extremely unlikely (≤3% probability). The precise borders of the box may vary somewhat, depending on where within the 15–30% range the threshold value is set. Treatment interventions based on this conceptual framework warrant validation in clinical trials.

**Table 2:** Prevalence of intra-abdominal candidiasis in different populations, and anticipated PPVs and NPVs of non-culture tests.

Prevalence (Reference)	Representative Patient			PCR					
		^1^ BDG, CAGTA		^2^ Leon et al. [8]		^3^ Nguyen et al. [13]		^4^ Fortun et al. [7]	
		PPV	NPV	PPV	NPV	PPV	NPV	PPV	NPV
5% [27,29]	- Low-to-moderate risk peritoneal dialysis patient with peritonitis	12%	97.6%	6%	97.7%	13%	98.9%	59%	99.2%
10% [28,44]	- Patient with emergent surgery for intra-abdominal infection - Patient with colonic perforation	22%	95%	12%	95.2%	24%	97.7%	76%	98.3%
20% [27,28]	- Patient (non-neutropenic) in SICU ≥ 7 days with abdominal surgery ^5^ - Patient with high-risk severe acute or necrotizing pancreatitis - Patient with small bowel perforation - Patient with emergent surgery for nosocomial intra-abdominal infection	39%	89.6%	24%	89.9%	41%	94.9%	88%	97.5%
30% [14,26,44]	- Patient who has undergone high-risk GI/hepatobiliary surgery - Patient with a biliary leak - Patient with a gastric/duodenal perforation - Patient (non-neutropenic) in SICU ≥ 7 days with abdominal surgery and *Candida* score >3 ^5^	53%	83%	35%	83.7%	55%	91.6%	93%	93.8%

Sensitivity and specificity of BDG and CAGTA are estimated as median values from the four studies of deep-seated candidiasis cited in the text. Sensitivity and specificity of PCR are estimated from the three studies of deep-seated candidiasis [7,8,13]. Sensitivity was rounded to 85% here for comparative purposes. There are no data on the performance of T2Candida for the diagnosis of deep-seated candidiasis, in the absence of candidemia. ^1^ Sensitivity/specificity: 65%/75%; ^2^ Sensitivity/specificity: 85%/33%; ^3^ Sensitivity/specificity: 85%/70% ^4^ Sensitivity/specificity: 85%/97%; ^5^ These patients may develop intra-abdominal candidiasis and/or candidemia. *Candida* score is a predictive model for invasive candidiasis that considers clinical variables, risk factors for candidiasis, and *Candida* colonization, which was developed by Leon et al. [44]. PCR: polymerase chain reaction; BDG: 1,3-β-d-glucan; PPV: Positive predictive value; NPV: Negative predictive value; GI: gastrointestinal. PPVs and NPVs within the dark black lines signify patients in whom non-culture testing may have greatest clinical utility, assuming that antifungal treatment is justified at a threshold likelihood of invasive candidiasis of ≥~15–30%. For these patients, a positive result is anticipated to move the likelihood of intra-abdominal candidiasis from below the threshold to above the threshold. At the same time, negative tests should assure that the likelihood of intrabdominal candidiasis is less than the threshold. The precise borders of the box may vary somewhat, depending on where within the 15–30% range the threshold value is set. Treatment interventions based on this conceptual framework warrant validation in clinical trials.
